# Supplementary material for: Determinants of early neonatal outcomes after emergency cesarean delivery at Hawassa University comprehensive specialised hospital, Hawassa, Ethiopia
Source: PLoS One. 2022 Mar 24;17(3):e0263837. doi: 10.1371/journal.pone.0263837 (PMC8947390; doi:10.1371/journal.pone.0263837)
Supplement: S1 Appendix — (DOCX) [file pone.0263837.s001.docx]

**SECTION 1: SOCIODEMOGRAPHIC CHARACTERISTICS**

101. How old are you?

------------Years

1. What is your religion?

1. Orthodox 4. Catholic

2. Muslim 5. Other

3. Protestant

1. What is your current marital status?

1. Married 3. Divorced

2. Unmarried 4. Widowed

104. From which region are you?

1. SNNPR 3. Other

2. Oromia

105. From which ethnic group are you?

1. Sidama 2. Oromo 3. Welayta

4. Halaba 5. Amhara 6. Gurage

7. Other

106. Where do you live?

1. Rural 2. Urban

107. Have you ever attended any school?

1. Yes 2. No

If the answer to question 107 is yes go to question number 108

108. What is your educational level?

1. Elementary school level 3. Diploma level

2. High school level 4. Degree level and above

109. What is your occupation?

1. Farmer 4. NGO employee

2. Merchant 5. House wife

3. Government employee 6. Others

110. Family income per month

­ _____________________ (Ethiopian birr)

**SECTION 2: REPRODUCTIVE CHARACTERSTICS**

201. Gravidity------------------ (write in number)

202. Parity ------------------- (write in number)

203. Gestational age

1. Preterm (< 37 weeks) 3. Post term (> 42 weeks)

2. Term (37 – 40 weeks)

204. Previous history abortion

1. Yes 2. No

If yes to Q 204 go to Q 205

205. Number of previous abortions

1. One 3. > Three

2. Two

206. Previous history of still birth

1. Yes 2. No

If yes Q 206, go to Q 207

207. Number of previous still births

1. One 3. >Three

2. Two

208. Previous history of early neonatal death

1. Yes 2. No

If yes to Q 208 go to Q 209

209. Number of previous early neonatal deaths

1. One 3. > Three

2. Two

210. Antenatal care in the current pregnancy

1. Yes 2. No

If yes to Q 210, go to Q211

211. Place of antenatal care

1. Health center/private clinic 3. Referral hospital

2. Primary hospital

212. History of previous uterine scar (cesarean or myomectomy)

1. Yes 2. No

If yes Q 212, go to Q 213

213. Number of previous uterine scars

1. One 2. Two 3. > Three

**SECTION 3: OBSTETRIC AND MEDICAL COMPLICATION**

301. Is there any maternal and/or fetal obstetric complication diagnosed per operatively?

1. Yes 2. No

If yes to question number 301 go to Q302

302. What type of ante partum obstetric complications identified?

1. Preeclampsia 6. Premature rapture of membrane

2. Eclampsia 7. Intrauterine growth restriction

3. Gestational hypertension 8. Oligohydramnios

4. Ante partum hemorrhage due to placenta previa 9. Others (Specify-----------------)

5. Ante partum hemorrhage due to abruption placenta

303. Is there any ante partum medical complication?

1. Yes 2. No

If yes to question number 303 go to question number 304

304. What was the type of ante partum medical disease or complications identified?

1. Diabetes mellitus 3. Anemia

2. Chronic hypertension 4. Cardiac disease

5. RVI 6. Other (specify-----------------)

**SECTION4: PREOPERATIVE AND INTRAOPERATIVE FETAL AND MATERNAL CONDITIONS**

401. Were you been at any health facility before arrival to this hospital labor and delivery ward?

1. No, I come directly from home 2. Yes, I was referred from health center

3. Yes, I was referred from governmental hospital

4. Yes, I was referred from private clinic/hospital

If she is referred, go to question number 402 and 403

402. How long it takes to reach this hospital from the last referring institution?

------------- Hrs

403. What was the diagnosis put as reason for referral?

1. Fetal tachycardia 6. Previous cesarean scar

2. Fetal bradycardia 7. Malpresentation

3. Obstructed labor 8. Cord prolapse

4. Ante partum hemorrhage 9. Prolonged second stage of labor

5. Preeclampsia/Eclampsia 10. CPD

11. Others (specify-----------------------)

404. Was she in labor preoperatively?

1. Yes 2. No

If yes to Q404, go to Q 405, Q406, Q407, Q408, and Q409

405. How was the onset of labor?

1. Spontaneous 2. Induced

406. For how long she labored before operation?

-------------------- Hours

407. What was the stage of labor during decision for operation?

1. Latent first stage of labor

2. Active first stage of labor3. Second stage of labor

408. What was the state of liquor at decision for operation?

1. Clear 4. Meconium stained and Grade three

2. Meconium stained and Grade one 5. Unknown

3. Meconium stained and Grade two

409. What was the presentation of the fetus?

1. Vertex 3. Face 5. Shoulder

2. Brow 4. Breech

410. What was the fetal heart beat at decision?

1. <120 beat per minute 3. > 160 beats per minute

2. 120 – 160 beats per minute

411. What was the indication for cesarean delivery?

1. Fetal tachycardia 8.Malpresentation

2. Fetal bradycardia 9.Failled instrumental delivery

3. Obstructed labor 10. Placenta previa in labor or with active bleeding

4. MSAF in LFSOL 11. Abruption of placenta with active bleeding

5. Cord prolapse 12. Previous uterine scar with x factor

6. CPD 13. More than one uterine scar in labor

7. Failed induction 14. Others (specify---------------)

412. What was the time of operation?

1. Working hours 2. Duty hours

413. What was the time interval between the decisions for operation to delivery of fetus?

------------------- Minutes.

414. What was the type of anesthesia?

1. Spinal anesthesia 2. General anesthesia

415. What was the blood pressure range of the patient during decision for operation, but prior to administration of Anesthesia?

1. < 100/60 mmHg 2. 100/60 – 139/89 mmHg

3. 140/90 mmHg and above

416. What was the blood pressure range of the patient after administration of anesthesia, but prior to fetal extraction?

1. < 100/60 mmHg 3. 140/90 mmHg and above

2. 100/60 – 139/89 mmHg

417. What was the type skin incision?

1. Pfannenstiel 2. Midline

418. What was the interval between skin incision to delivery of the fetus?

------------------- Minutes.

419. What was the position of the surgeon?

1. Year one resident 4. Year four resident

2. Year two resident 5. Senior

3. Year three resident

**SECTION 5: NEONATAL CLINICAL CONDITION**

501. What is the sex of neonate?

1. Male 2. Female

502. What was the weight of the neonate?

1. Low birth weight (<2500 grams) 3. Large birth weight (> 4000 grams)

2. Normal birth weight ( 2500-3999 grams)

503. Is there any positive sign of life during extraction?

1. Yes 2. No

If yes to Q 503, proceed with subsequent questions

504. What was the first minute Apgar score?

1. < 7 2. >7

505. What was the fifth minute Apgar score?

1. < 7 2. > 7

506. Was the neonate referred to NICU immediately after delivery?

1. Yes 2. No

If yes to Q 506, go to Q 507, and Q 508

507. What was the neonatal diagnosis during referral?

1. Preterm 4. PNA

2. Low birth weight 5. RDS

3. MAS 6. Others (-------------------)

508. For how long the neonate stayed at NICU?

1. < 24 hours 2. > 24 hours

509. What was neonatal condition on the last seventh post operation days?

1. Discharged improved 3. Died

2. Alive on treatment

If died to Q 509 go to Q 510 and Q 511

510. What was cause of death?

1. PNA 3.MAS 5. Others

2. RDS 4.EONS

511. On which post operation day was the neonate died

1. First 4. Fourth 7. Seventh

2. Second 5. Fifth

3. Third 6. Sixth
