# Supplementary material for: Determinants of early neonatal outcomes after emergency cesarean delivery at Hawassa University comprehensive specialised hospital, Hawassa, Ethiopia
Source: PLoS One. 2022 Mar 24;17(3):e0263837. doi: 10.1371/journal.pone.0263837 (PMC8947390; doi:10.1371/journal.pone.0263837)
Supplement: Attachment — Submitted filename: response to second reviewer.docx [file pone.0263837.s003.docx]

Response for the second reviewer

1. I humbly accept all General comments	and they all are corrected

Reference number 27…mentioned at the sample size estimation part

2. **Abstract**: Comments are Accepted and corrected
3. **Introduction**: Classification of the cesarean section depends on different factors like Emergent or non-emergent cesarean sections and is based on women’s request in some countries which is not accepted in our country unless the Mather had cesarean section scar. The other classification is the Robson classification of a cesarean section which is recommended by WHO.

- The reference is number is 11

4. **Methods:** comments are accepted

- Negative fetal heartbeat: intrauterine fetal death cases at admission were excluded
- Those babies, who were healthy, were usually discharged after 24 hours. Since the study includes the first 7 days, we just contacted the mother on the phone whether the baby is breastfeeding well, alert, sleeping well, having fast breathing, and fever. We do this only for healthy babies but if there is any problem, the mother was informed to bring it back.
- The Mather was informed about the study, that there will be follow up upon discharge till 7 days, while she is in the hospital
- Discharged neonates were followed by data collectors assigned to specific neonates.

5. **Results:** comments are accepted

- Cause of neonatal death were taken from neonatal intensive care unit registration book and we don’t have set up to autopsies so we commonly reach the conclusion by clinical characteristics

6. **Discussion:** comments are accepted and corrected
7. **Consent:** comments are accepted and corrected
8. **References:** comments accepted and corrected
